# Prevalence of Tobacco Use Among Rural-Dwelling Individuals Who Inject Drugs

**DOI:** 10.1001/jamanetworkopen.2020.0493

**Published:** 2020-03-10

**Authors:** Wajiha Z. Akhtar, Marlon P. Mundt, Ruth Koepke, Sarah Krechel, Michael C. Fiore, David W. Seal, Ryan P. Westergaard

**Affiliations:** 1 Department of Medicine, University of Wisconsin School of Medicine and Public Health, Madison; 2 University of Wisconsin Center for Tobacco Research and Intervention, Madison; 3 Wisconsin Department of Health Services, Madison; 4 Vivent Health, Milwaukee, Wisconsin; 5 Tulane University School of Public Health and Tropical Medicine, New Orleans, Louisiana

## Abstract

This cross-sectional study assesses the prevalence of smoking through a multisite survey of individuals aged 15 years or older living in rural Wisconsin who inject drugs.

## Introduction

The overall adult prevalence of smoking is at a historic low in the US, in large part because of effective policy interventions, youth prevention efforts, and evidence-based cessation interventions for current smokers.^1,2^ However, tobacco use rates remain high among subpopulations defined primarily by educational level, socioeconomic status, and the presence of comorbid mental health and substance use diagnoses.^3^ The ongoing epidemic of opioid use disorder has particularly affected rural communities, and recent outbreaks of HIV have led to the recognition that rural-dwelling people who inject drugs (PWID) are a population with unique health needs.^4^ To understand the potential burden of tobacco-related illness in this population, we assessed the prevalence of smoking through a multisite survey of PWID in rural Wisconsin.

## Methods

In this cross-sectional study, we recruited participants using respondent-driven sampling^5^ at 6 syringe service program offices in Wisconsin from January 2018 to July 2019 as part of a larger study assessing unmet health care needs among PWID. Eligible participants were aged 15 years or older, reported injecting drugs to get high in the past 30 days, and resided within the service areas of the syringe service programs in rural central and northern Wisconsin. Current smoking status was obtained via self-report using audio computer-assisted self-interview. All respondents provided written informed consent and were compensated $20 in cash for completing the survey and an additional $10 for each peer referred to the study. The study protocol was approved by the institutional review board of University of Wisconsin–Madison. This study followed the Strengthening the Reporting of Observational Studies in Epidemiology (STROBE) reporting guideline.

We estimated the prevalence of smoking and then compared participant characteristics between those who smoked cigarettes and those who did not using the χ^2^ test for categorical variables and the Wilcoxon rank sum test for continuous variables. Statistical significance was set at a 2-tailed *P* < .05, and analysis was performed using SAS version 9.4 (SAS Institute).

## Results

Cigarette use data were available for 986 of 993 participants (99.3%). A total of 909 (92.2%) reported that they currently smoked cigarettes. The median (interquartile range) age was 33 (27-40) years, 580 (58.9%) were men, and 786 (80.2%) were white, non-Hispanic participants (Table). Compared with participants who were not currently smoking cigarettes, those who reported smoking were more likely to be enrolled in Medicaid (29 [58.0%] vs 455 [74.2%]; *P* = .04) and more likely to have experienced homelessness in the last 6 months (33 [45.2%] vs 561 [64.0%]; *P* = .002). Less than half of the participants (394 [42.0%]) reported receiving any medical care from a primary care physician in the prior 6 months.

**Table.** Participant Characteristics by Current Cigarette Smoking Status

Characteristic	No. (%)			*P* Value^a^
	Cigarette Smoking Status		Total (N = 986)	
	Current (n = 909)	Noncurrent (n = 77)		
Age, median (IQR), y	33 (27-40)	34 (26-40)	33 (27-40)	.82
Men	537 (59.1)	43 (55.8)	580 (58.8)	.19
Race/ethnicity				
White, non-Hispanic	721 (79.8)	65 (84.4)	786 (8.2)	.50
Native American	102 (11.2)	8 (10.4)	110 (11.1)	
Other	81 (9.0)	4 (5.2)	85 (8.7)	
Education				
<High school diploma	161 (17.8)	12 (15.6)	173 (17.6)	.08
High school diploma or GED	426 (47.0)	28 (36.3)	454 (46.1)	
>High school diploma	320 (35.3)	37 (48.1)	357 (36.3)	
Had health insurance coverage	643 (70.7)	53 (68.8)	696 (70.6)	.91
Insured by Medicaid^b^	455 (74.2)	29 (58.0)	484 (73.0)	.04
Attended a primary care visit in the past 6 mo^c^	358 (41.4)	36 (48.7)	394 (42.0)	.23
Experienced homelessness in the past 6 mo^d^	561 (64.0)	33 (45.2)	594 (62.5)	.002
Drug of choice				
Methamphetamine	472 (52.4)	42 (56.8)	514 (52.8)	.22
Heroin	287 (31.9)	19 (25.7)	306 (31.4)	
Cocaine	26 (2.9)	0	26 (2.7)	
Other	115 (12.8)	13 (17.6)	128 (13.1)	
Binge drank at least once in the past 30 d^e^	544 (59.9)	39 (50.7)	583 (59.1)	.12
Average packs of cigarettes smoked per day				
<Half a pack	167 (20.7)	NA	167 (2.7)	NA
Half pack to 1 pack	359 (44.5)	NA	359 (44.5)	
≥1 pack	281 (34.8)	NA	281 (34.8)	
Hepatitis C reactive	318 (35.0)	29 (37.7)	347 (35.2)	.38

Abbreviations: IQR, interquartile range; GED, general education diploma; NA, not applicable.

^a^ From χ^2^ test for heterogeneity for categorical variables and the Wilcoxon rank sum test for continuous variables.

^b^ The denominator includes 663 participants who reported having health insurance.

^c^ Participants who reported receiving care from a private physician or a community health center were considered receiving primary care.

^d^ The denominator includes 950 participants who responded to the question.

^e^ Binge drinking was assessed as 5 or more drinks containing alcohol per binge-drinking episode for men and 4 or more drinks containing alcohol per binge-drinking episode for women.

## Discussion

Among this large sample of rural-dwelling individuals who were actively injecting opioids and/or stimulants, an extremely high proportion reported that they currently smoked cigarettes. The smoking rate of more than 90% is among the highest reported among any subpopulation and exceeds that of many other groups with a high prevalence of smoking, including adults with schizophrenia.^1,2,6^ In contrast, the overall adult smoking prevalence rate in the US in 2018 was 13.8%.^1,6^

Although tobacco control interventions during the past several decades have led to overall declines in smoking, PWID in rural communities appear not to have been reached by these measures. The health risks associated with tobacco use among rural-dwelling PWID may be exacerbated by limited access to primary care, unstable housing, and other social determinants. Our findings suggest that novel approaches to eliminating tobacco use among this population are needed.

Limitations of this study include its focus on residents of a single US state and reliance on a nonrandom sampling method. Respondent-driven sampling, a social network–based recruitment strategy, is a widely used tool for engaging difficult-to-reach populations in research, but it may underestimate the variability within populations because of the tendency of participants to recruit peers with similar characteristics. However, preliminary analysis suggests that there were no differences in smoking prevalence based on whether participants recruited their peers to the study. Network chains of participants recruited by their peers did show variability in terms of drug of choice, syringe sharing behavior, and alcohol use. Future research should examine regional and sociodemographic variability of tobacco use patterns among PWID and seek to inform smoking cessation interventions that are appropriately tailored to the health and social service needs of this population.
